# Reduced Intellectual Development in Children with Prenatal Lead Exposure

**DOI:** 10.1289/ehp.8552

**Published:** 2005-12-29

**Authors:** Lourdes Schnaas, Stephen J. Rothenberg, Maria-Fernanda Flores, Sandra Martinez, Carmen Hernandez, Erica Osorio, Silvia Ruiz Velasco, Estela Perroni

**Affiliations:** 1 National Institute of Perinatology, Mexico City, Mexico; 2 National Institute of Public Health, Cuernavaca, Morelos, Mexico; 3 Centro de Investigaciones y de Estudios Avanzados-Instituto Politécnico Nacional, Merida, Yucatan, Mexico; 4 Institute for Research in Applied Mathematics and Systems, National Autonomous University of Mexico, Mexico City, Mexico

**Keywords:** child development, intelligence, lead, prenatal exposure delayed effects

## Abstract

**Objective:**

Low-level postnatal lead exposure is associated with poor intellectual development in children, although effects of prenatal exposure are less well studied. We hypothesized that prenatal lead exposure would have a more powerful and lasting impact on child development than postnatal exposure.

**Design:**

We used generalized linear mixed models with random intercept and slope to analyze the pattern of lead effect of the cohort from pregnancy through 10 years of age on child IQ from 6 to 10 years. We statistically evaluated dose–response nonlinearity.

**Participants:**

A cohort of 175 children, 150 of whom had complete data for all included covariates, attended the National Institute of Perinatology in Mexico City from 1987 through 2002.

**Evaluations/Measurements:**

We used the Wechsler Intelligence Scale for Children–Revised, Spanish version, to measure IQ. Blood lead (BPb) was measured by a reference laboratory of the Centers for Disease Control and Prevention (CDC) quality assurance program for BPb.

**Results:**

Geometric mean BPb during pregnancy was 8.0 μg/dL (range, 1–33 μg/dL), from 1 through 5 years was 9.8 μg/dL (2.8–36.4 μg/dL), and from 6 through 10 years was 6.2 μg/dL (2.2–18.6 μg/dL). IQ at 6–10 years decreased significantly only with increasing natural-log third-trimester BPb (β = −3.90; 95% confidence interval, −6.45 to −1.36), controlling for other BPb and covariates. The dose–response BPb–IQ function was log-linear, not linear–linear.

**Conclusions:**

Lead exposure around 28 weeks gestation is a critical period for later child intellectual development, with lasting and possibly permanent effects. There was no evidence of a threshold; the strongest lead effects on IQ occurred within the first few micrograms of BPb.

**Relevance to Clinical Practice:**

Current CDC action limits for children applied to pregnant women permit most lead-associated child IQ decreases measured over the studied BPb range.

Prospective lead studies of child development from the 1980s to date show associations between low blood lead (BPb) concentration and poor neurobehavioral development (Baghurst et al. 1992; Bellinger 1989; Bellinger et al. 1986, 1987, 1994; Bornschein et al. 1985; Dietrich 1991; Dietrich et al. 1987a, 1987b, 1991, 1993a, 1993b; McMichael et al. 1988, 1992; Schnaas et al. 2000; Wasserman et al. 1992, 1994, 1997, 2000a, 2000b), although the focus of most of these studies has been postnatal exposure. Only some studies included measurement of maternal BPb during pregnancy or at delivery (Bornschein et al. 1985; Graziano et al. 1990; Rothenberg et al. 1994). A Yugoslavia study (Wasserman et al. 2000a) used a repeated-measures design and found that increased mid-pregnancy BPb (12–20 weeks) was significantly associated with decreased 3- to 7-year intelligence quotient (IQ) regardless of pattern of postnatal exposure. A Cincinnati, Ohio (USA), study (Ris et al. 2004) showed lasting significant effects of BPb between 6 and 28 weeks on factor scores representing attention and visuoconstruction in adolescents when prenatal BPb was tested without simultaneously considering postnatal BPb exposure history.

Ideally, we would like to include the entire history of lead exposure in assessing lasting effects of lead on child development. When the study sample is exposed to relatively constant sources of environmental lead, there is often substantial tracking of BPb over time (Tong et al. 1996; Wasserman et al. 2000a), producing high correlations among serial BPb levels between and within prenatal and postnatal periods. Collinearity among highly correlated BPb variables in the same linear model will produce biased estimates of lead effect with inflated SEs. On the other hand, piecemeal analysis of lead effects, testing one period of lead exposure at a time, ignores potential effects of earlier or later exposure. Such omission could lead to residual confounding of tested lead effects.

The principal lead exposure sources in pregnant women and their children in the Mexico City Prospective Lead Study were air lead and lead from ceramic ware (Schnaas et al. 2004). Air lead decreased continually throughout the 15-year study period because of reduction and elimination of lead in gasoline. Individual exposure to leaded ceramic ware was both idiosyncratic and intermittent. Such variable individual lead exposure substantially reduced BPb tracking in this sample and allowed an analysis of the effect of lead exposure from 12 weeks of pregnancy through the first 10 years of life on child intelligence from 6 to 10 years of age.

## Materials and Methods

### Subjects.

The subjects belonged to a cohort of children born in Mexico City at the National Institute of Perinatology between 1987 and 1992, followed until 2002. The Ethics Committee of the National Institute of Perinatology approved the research protocol. Investigators met with parents, verbally explained the project, and asked them to read the description in the informed consent and to sign if they wished to participate with their child. We recruited women at 12 weeks of pregnancy and measured BPb every 8 weeks to delivery. We also measured BPb from maternal and cord blood at delivery. A total of 321 children born to these women met the following inclusion criteria: child born with at least 36 weeks of gestational age, 5-min Apgar score ≥ 6, birth weight > 2,000 g, without major or minor congenital anomalies or being the product of multiple birth. We evaluated children with psychometric tests, anthropometric measurements, and BPb at 6-month intervals after birth. We collected data on demographic, socioeconomic, and other factors that might constitute potential confounders or important control variables modifying the relationship between lead and child development.

Of the 321 infants comprising the original sample, we successfully tested 175 children after 5 years of age.

### BPb measurements.

Venous blood was drawn into purple-top Becton-Dickinson Vacutainers (Franklin Lakes, NJ, USA) with EDTA anticoagulant. Environmental Science Associates Laboratories Inc. (Chelmsford, MA, USA) determined BPb in duplicate analysis by anodic stripping voltammetry. Samples with mean duplicate values < 5 μg/dL were reanalyzed via atomic absorption spectrometry. Quality control information is provided elsewhere (Rothenberg et al. 1994). The lab is a reference laboratory for the Centers for Disease Control and Prevention (CDC) Blood Lead Laboratory Reference System (Atlanta, GA, USA) and participates in the Commonwealth of Pennsylvania Department of Health Blood Lead Proficiency Testing Program (Exton, PA, USA). BPb during pregnancy was measured every 8 weeks starting at week 12 of pregnancy. We used the geometric mean of lead at 12 and 20 weeks as the lead measure for the second trimester of pregnancy and the geometric mean of lead at 28 and 36 weeks as the lead measure for the third trimester of pregnancy. We calculated geometric mean BPb from biannual measurements from 6 months to 5 years and used BPb at each age from 6 to 10 years to measure lead exposure contemporaneous with each year’s IQ measurement. In supplementary models, we also used maternal BPb at each prenatal measurement and postnatal geometric mean yearly BPb from 1 to 5 years.

### Developmental assessment.

We assessed child intelligence under standardized conditions with the Wechsler Intelligence Scale for Children–Revised (WISC-R; Spanish version) (Wechsler 1981a), providing a Full-scale IQ (FSIQ). The WISC-R has 12 subtests, six of which are used to estimate a Verbal IQ (VIQ), and the remaining six a Performance IQ (PIQ). Three psychologists unaware of child BPb evaluated IQ. For each child evaluated by a psychologist, the two other psychologists reviewed the test protocols and assigned scores given by the examiner for each test. An analysis of variance with post hoc testing for mean IQ grouped by examining psychologist and child age was used to assess possible psychologist bias.

### Covariates.

We measured maternal IQ with the Wechsler Adult Intelligence Scale (Spanish) (Wechsler 1981b). We constructed an index for socioeconomic status (SES) based on head of household education and occupation, and family income. We evaluated degree of stimulation and quality of caretaker–child interaction in the home environment using the HOME Scale (Home Inventory for Families of Infants and Toddlers) (Caldwell and Bradley 1984). All covariates used in statistical analyses were collected during pregnancy or in the first 6 postpartum months.

### Data analyses.

We used Fisher exact tests, Pearson chi-squared with exact probability, or *t*-tests to contrast subjects included in the analysis, subjects lost to follow-up in the first 5 postnatal years, and subjects with incomplete post-5-year data with the variables sex, SES, BPb at different ages, maternal IQ and educational level, and postnatal developmental scores.

Descriptive statistics, identification of outliers, and appropriate transformations were performed before bivariate and multivariate analyses. BPb was converted to natural logarithms to eliminate heteroskedasticity and normalize skewed distributions of residuals, reduce the influence of outlying high lead values on regression coefficients, and adequately specify the functional relation of BPb on FSIQ. We examined associations between the FSIQ measured from 6 to 10 years of age and each measurement of lead exposure in panel regression analyses (Kennedy 2003), first without covariates and then controlling by child sex, SES, maternal IQ, HOME, and birth weight, instead of bivariate regressions, to adjust the regressions for repeated measurements of IQ in each subject.

Because the data are multilevel with BPb at each age nested within children, we used a linear mixed model to analyze the pattern of lead effect on FSIQ evaluated from 6 to 10 years of age. The dependent variable was FSIQ at ages 6, 7, 8, 9, and 10, whereas the independent variables with fixed effects were maternal IQ, child sex, SES, birth weight, geometric mean of BPb during the first 5 years of age, BPb at each age at which the FSIQ measurements were made, geometric mean of BPb during the second and third trimester of pregnancy, and a dummy variable indicating the first FSIQ measurement of the child, allowing control for test learning between the first IQ measurement and the following ones. Some children had their first WISC-R test at 6 years of age; others were first tested at 7 or 8 years.

We included as random effects subject and BPb measured at each year of WISC-R FSIQ measurement of the child. We modeled the covariance matrix of the residual error by a first-order autoregressive process. We used the likelihood ratio test to determine if the addition of random intercepts, random slopes, and autoregressive residual covariance matrix significantly improved model fit.

To examine the effect of simultaneous inclusion of all lead variables, we constructed several mixed models each with only one lead variable and statistically compared those lead coefficients with the lead coefficients of the mixed model with all lead variables. We also constructed mixed models without the control variables to determine lead coefficients unadjusted for covariates. We performed the same analyses for VIQ and PIQ.

We used the Bayesian Information Criterion (Hardin and Hilbe 2001) to determine which model best fit the data. The information criterion includes a penalized function based on number of estimated parameters. If the number of parameters increases without substantial model improvement, the information criterion also increases, indicating a poorer data fit.

The two-level model had two different residuals: level-1 residuals, annual observations calculated by subtracting the linear predictor from the FSIQ; and level-2 residuals, the empirical Bayes predictions considered as higher-level residuals.

To check the normality of the two residual types, we generated kernel density plots with overlaid normal density functions and plotted quartiles of the residuals against quartiles of a normal distribution to emphasize possible non-normality near the tails. Shapiro-Wilk and Shapiro-Francia tests were also used to check residual normality. We divided level-2 residuals by the SEs (from the posterior SDs) to detect outliers and plotted residuals against predictions to examine homoskedasticity.

In addition to calculating correlations among BPb variables to assess potential for collinearity, we also ran an artificial multiple regression with the full mixed-model variables to calculate the variance inflation factors (VIFs) (Hardin 1995) for the lead terms. As a final check on the possibility that collinearity among lead variables significantly affected the pattern of results in the mixed model, we converted the group of lead variables to orthogonal variables and ran the model again.

We refit the mixed models with linear lead terms and used the *J*-test (MacKinnon 1981) to determine if the logarithmic specification of the lead variables produced a better fit to the data than a linear lead specification.

## Results

From the sample of 175 children retained to 6 years of age, we studied 150 with complete data for all covariates included in the model. There were no significant differences in sex, SES, birth weight, FSIQ of the child, maternal IQ, BPb at second trimester, and geometric mean BPb from 1 to 5 years of age between children included and not included in the analyses (Table 1). BPb in the third trimester of pregnancy and at 9 and 10 years of age was significantly lower in the group with complete data.

More of the group dropping out before reaching 6 years tended to be in the lowest SES, compared with the tested group with complete data. Bayley (1969) and McCarthy (1972) developmental scores also tended to be lower for this group.

**Figure 1:**
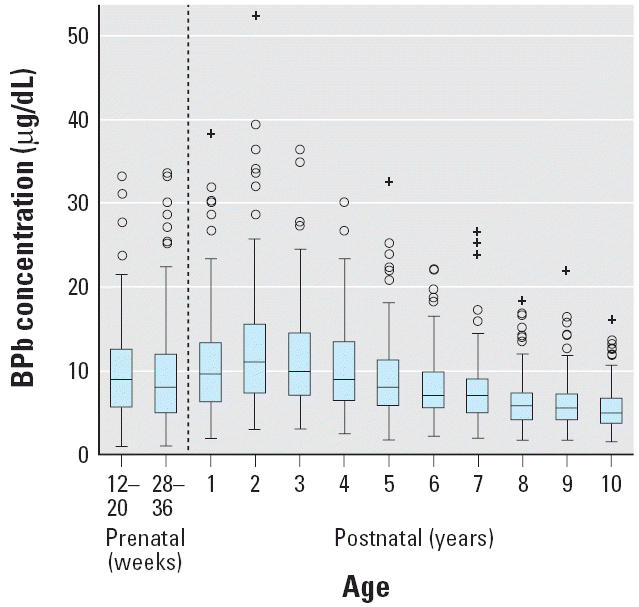
Box plots of BPb by age of the 150 children sampled for the linear mixed-model analyses. The lower and upper limits of the rectangular boxes indicate the 25th to 75th percentile range, and the horizontal line within the boxes is at the 50th percentile. The vertical lines extending from the bottom and top of the boxes represent lead values 1.5 times the interquartile range below and above the 25th and 75th percentile, respectively. Open circles represent lead values between 1.5 and 3.0 times the interquartile range below and above the 25th and 75th percentile, and pluses indicate lead values exceeding 3.0 times the interquartile range limit. Plots for each age, with extreme high values, are typical for log distributions plotted on a linear scale. Conversion factor: 10 μg/dL = 0.483 μmol/L.

The Pearson correlation between the 12-to 20-week and 28- to 36-week prenatal natural log BPb was 0.48, between either of the prenatal and any of the postnatal BPb ≤ 0.23, and between the 1- to 5-year and 6- to 10-year postnatal BPb = 0.70. VIFs for all variables in the model were < 2.2 (mean VIF = 1.45), where VIF ≥ 10 is considered significant (Chaterjee et al. 2000). Models using orthogonal lead variables showed no change in model results. Collinearity among simultaneously included lead variables in the models was not a factor in the results presented below. Figure 1 shows the distribution of BPb from the cohort followed to 6–10 years of age.

Fixed-effects panel regression analyses, unadjusted for covariates, testing separate pre-natal, perinatal, and postnatal BPb with FSIQ of the child showed IQ reduction associated with BPb increase for all lead measurements (Table 2). However, the only significant BPb effects were with BPb at third trimester of pregnancy and BPb measured simultaneously with IQ tests.

We performed panel analyses for FSIQ with each of the other covariates alone. All covariates were associated with IQ in the expected direction, but only maternal IQ (*p* < 0.001), maternal educational level (*p* < 0.001), SES (*p* < 0.01), and HOME score (*p* < 0.05) showed positive significant effects on the FSIQ of the child (analyses not shown).

Using a linear mixed model with random intercept and random slope for 6- to 10-year BPb and adjusting for all covariates (Table 3, model A), we found that children whose mothers had higher BPb during 28–36 weeks of pregnancy had significantly lower FSIQ, children of the higher IQ mothers had higher FSIQ at all ages, and child FSIQ in the first evaluation was significantly lower than in subsequent evaluations. Most other remaining covariates were associated with child intelligence in the expected direction but were not significant.

We developed additional models containing all prenatal and postnatal BPb with only the covariates that were significant in the full linear mixed model (maternal IQ and the dummy variable for the first FSIQ measurement of the child). Nonsignificant BPb variables were eliminated, so the model then included only significant covariates (Table 3, model D). The third-trimester BPb coefficients in all additional models with progressive deletion of nonsignificant variables (Table 3, models B, C, and D) were not significantly different from the coefficient found in the full mixed model (Table 3, model A), also suggesting minimal collinearity effects.

In the linear mixed model with VIQ as the dependent variable, higher 28- to 36-week BPb was associated with lower intellectual coefficient of the child (β = −3.15, *p* = 0.007), and children of higher IQ mothers had better performance (β = 0.29, *p* < 0.001). There was no significant change in the VIQ from the first evaluation to subsequent evaluations.

In the linear mixed model with PIQ as the dependent variable, 28- to 36-week BPb was inversely associated with child IQ (β = −4.37, *p* = 0.004), and children of the higher IQ mothers had better performance (β = 0.40, *p* < 0.001). We also observed significant PIQ improvement from first to subsequent tests (β = 7.2, *p* < 0.001).

To evaluate more precisely at which pregnancy stage maternal BPb was best associated with later reduction in child IQ, we constructed a linear mixed model adjusted by the same covariates used in the full linear mixed model (Table 3, model A) but exchanged the averaged prenatal BPb variables for prenatal BPb at week 12, 20, 28, or 36 of pregnancy. BPb at week 28 of pregnancy was the only prenatal lead measure significantly predicting lower FSIQ (β = −4.13, *p* < 0.001) (Table 4).

To statistically test whether natural log BPb transformation fit the data significantly better than a linear BPb specification, we used the *J*-test to compare the two specifications of lead at the third trimester of pregnancy on FSIQ in the full model (Table 3, model A). The logarithmic form of third-trimester BPb fit the data significantly better than did a linear functional form (*t* = 2.15, *p* = 0.02).

**Figure 2:**
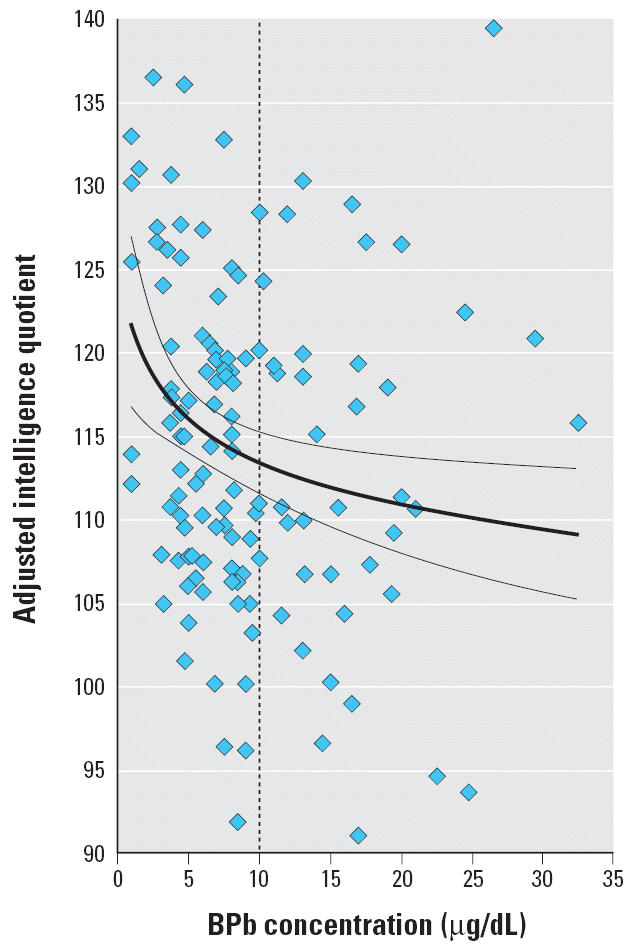
Partial residual plot of the effect of third-trimester maternal BPb (thin lines: 95% confidence interval) on FSIQ at 8 years of age adjusted for maternal IQ, sex, birth weight, SES, and BPb levels at other prenatal and postnatal ages. Conversion factor: 10 μg/dL = 0.483 μmol/L.

Figure 2 shows a partial residual plot of the effect of third-trimester maternal BPb on FSIQ at 8 years of age adjusted for the covariates and other BPb values in the full model (Table 3, model A).

## Discussion

Increased maternal lead concentration at third trimester of pregnancy, especially around week 28, was associated with decreased intellectual child development, even after controlling for other prenatal and postnatal lead measurements. Other studies found significant adverse associations between postnatal BPb and IQ (Baghurst et al. 1992; Bellinger et al. 1992; Dietrich et al. 1993b; Wasserman et al. 1997). In our panel unadjusted regression analyses, we noted a significant effect of 6- to 10-year BPb on child IQ as well, but this effect lost significance when other BPb and covariates were included in mixed-model analysis. Collinearity between prenatal and 6- to 10-year BPb variables was not responsible for loss of explanatory power of 6- to 10-year BPb, as shown in the extensive diagnostic testing reported in “Results.” Given the modest sample size and relatively low power of this study, we do not claim that lead exposure from 6 to 10 years or any other developmental period has no effect on child IQ. More likely, third-trimester lead exposure is a more powerful predictor of later child IQ and absorbed enough of the variation in IQ formerly attributed to 6- to 10-year BPb to render it insignificant in our model.

In contrast to other studies in which prenatal lead exposure biomarkers were umbilical cord BPb (Bellinger et al. 1992) or one (Dietrich et al. 1993b; Ris et al. 2004; Wasserman et al. 1997, 2000a) or at most two (Baghurst et al. 1992) maternal lead measurements during pregnancy, we measured prenatal lead exposure systematically (within an interval of ± 2 weeks) during specific pregnancy stages (weeks 12, 20, 28, and 36 of pregnancy, at delivery, and in umbilical cord). We note that 28-week fetal central nervous system development is distinctly different than development either at 12 weeks or at term. Neuroblast proliferation is essentially complete before 28 weeks, whereas neuronal migration and aggregation continue through the first half of the third trimester. Myelination of tracts within the developing human fetal brain has just begun by 25 weeks (Herschkowitz 1988). Deeper cortical layers are poorly defined at 24 weeks, clearly developed at 28 weeks, and reach postnatal appearance by 34 weeks of pregnancy (Larsen 1997). Limiting the range of permitted weeks of pregnancy for placing each maternal BPb in its nominal category probably enhanced our ability to detect pregnancy phase-specific BPb effects.

Other studies did not simultaneously include all lead measurements in their analyses, although one (Wasserman et al. 2000a) included directional postnatal lead change indicators along with the single pregnancy BPb variable. We were able to include the entire history of lead exposure in our analyses because collinearity among the lead measures was not a significant factor. In our analysis, simultaneous inclusion of 6- to 10-year BPb and the remaining BPb reduced the size of the 6- to 10-year lead coefficient without changing its variance, rendering it insignificant. With the increased power afforded by a larger sample size, 6- to 10-year BPb might well have retained its significance.

### Methodologic considerations.

A frequent problem in cohort studies is high loss rate during extended follow-up. From an original sample of 321 children, we tested 175 available children after 5 years of age, of which only 150 were included in mixed-model analyses due to missing covariates. The smaller number of subjects reduced the possibility of detecting subtle effects and increased the possibility of instability of model coefficients. Nevertheless, despite the medium sample size, we found a highly significant effect of maternal third-trimester BPb on child IQ at 6–10 years of age with little evidence of selective dropout bias in the descriptive statistics.

In addition to longitudinal analyses, we carried out separate analyses of IQ at each age. The pattern of results was consistent in these analyses; 28- to 36-week BPb and maternal intellectual quotient were the variables significantly predicting child IQ.

Repeated use of the same test to evaluate child IQ at short intervals could lead to learning of test components across time and a familiarization with the test situation. We found a significant change (Table 3) only between the first FSIQ measurement and the subsequent measurements, nearly all of which was due to increase in the performance scale. Repeated test application produced a significant adjusted increase of 7.2 PIQ points and 4.0 FSIQ points from first to subsequent test applications. This might be expected in children encountering performance tasks for the first time during the initial test application.

Studies in developmental toxicity have shown that subtle developmental alterations are easier to detect when subjects confront challenging or stressful situations (Cory-Slechta 1990; Rice and Baron 2000). Familiarity with the test situation and repetition of the same test should have reduced our ability to detect subtle developmental deficits associated with lead. We conclude that the lead effect described is robust.

Other studies found a substantial impact of sociodemographic variables on IQ. Several studies reported significant associations between lead and child development that disappear (Ernhart et al. 1989) or become evident (Wasserman et al. 1997) when HOME score was used as a covariate. We applied the 6-month HOME Scale in our study but did not include it in full modeling because it did not appreciably or significantly change the estimated magnitude and significance of BPb and because model fit improved according to the Bayesian Information Criterion (Hardin and Hilbe 2001) when this covariate was omitted (see Appendix for mixed models with HOME added). In contrast to our models, other studies found the HOME Scale useful. The HOME Scale is distinctly Euro-North American culture bound. For example, we found that many homes we visited did not have items such as educational toys, which were not readily available in the domestic market at that time, thus altering the HOME score of our subjects. Furthermore, 6-month evaluations might be expected to play little role in development at 6–10 years.

If increased third-trimester BPb levels were associated with decreased birth weight and low birth weight was associated with poor postnatal intellectual development, the modeled effect of third-trimester BPb on 6- to 10-year IQ could be mediated through lead effect on birth weight. Our subject inclusion criteria accepted only newborn infants into the study with birth weight > 2,000 g (the Mexican standard for low birth weight at the time of the study), thus excluding cases with the highest probability of showing later deficits due to low birth weight. Exploratory modeling showed that no prenatal BPb was significantly associated with birth weight. Finally, excluding or including birth weight in mixed-model analyses changed the 28- to 36-week BPb coefficient by less than 0.03. There is no evidence in these data that third-trimester BPb effect on 6- to 10-year-old IQ was mediated by lead effect on weight.

Both the Spanish WISC-R for child IQ and the Spanish Wechsler Adult Intelligence Scale used for maternal IQ have since been superseded by updated, renormalized versions. The tests we used were the only Spanish language versions available during data collection. We note that the IQ scores measured in our sample were generally higher than those obtained in other prospective studies, perhaps as a result of using outdated tests. Although the current version of the WISC might reveal the bias in the absolute IQ associated with lead in these data, the covariation between BPb and IQ was likely not affected by the specific test version used.

### Public health implications.

These data suggest that the early third trimester of pregnancy may constitute a critical period for subsequent intellectual child development, during which lead exposure can produce lasting and possibly permanent effects. In addition, the data suggest there is no threshold for the adverse consequences of lead. On the contrary, the largest IQ changes in our sample are observed within the first few micrograms per deciliter of BPb—that is, at lower BPb (Figure 2). The relationship between BPb and child IQ is logarithmic, not linear, as shown by the significant (*p* = 0.02) *J*-test. Other studies have already reported larger IQ change with change of lead at lower concentrations than at higher concentrations (Canfield et al. 2003; Lanphear et al. 2000; Schwartz 1994). A recent reanalysis of a large (*n* = 1,333) pooled data analysis (Lanphear et al. 2005) of seven prospective lead studies, including this one, also confirms that the log-linear dose–response relationship between IQ around 7 years of age and contemporary BPb is superior to a linear–linear dose–response relationship (Rothenberg and Rothenberg 2005).

We noted the same pattern of BPb change during pregnancy in this study (Rothenberg et al. 1994) observed in other studies in the United States (Hertz-Picciotto et al. 2000; Schell et al. 2000). Postnatal BPb pattern with age has already been examined in detail in this cohort (Schnaas et al. 2004) and is similar to that from United States and Australian prospective studies (Dietrich et al. 1991; McMichael et al. 1988). Postnatal BPb peaks around 2 years of age and then decreases with increasing age (Figure 1). Because our cohort did not exhibit unusual BPb change from 12 weeks of pregnancy through 10 years of age, our results cannot be attributed to the cohort’s unique history of lead exposure.

Across a range of BPb from 1 to 32 μg/dL, these data show that half of the deleterious effects of lead on child IQ measured here occurred when third-trimester BPb increased from 1 to 6 μg/dL. When maternal BPb reached current Mexican and U.S. action limits for children and pregnant women (10 μg/dL), most of the adverse consequences on later child IQ associated with lead in the measured range had already occurred. If we continue to accept the current action limit, we also accept that most of the “damage” to the IQ of children associated with third-trimester lead exposure in our sample is permissible.

The fetal brain seems susceptible to lower lead concentrations than those established by the official Mexican standard and current CDC guidelines, and the effects are obvious at least until 10 years of age. Although these findings should be replicated, our data suggest that we should establish lower action limits for lead exposure of reproductively active women.

## Figures and Tables

**Table 1:** Comparison among subjects included in the model and subjects with incomplete data, or not assessed beyond 5 years of age.

	Not assessed beyond 5 years of age		Subjects with incomplete data		Subjects included in the model			
Characteristics	No. (%)	BPb GM (5th–95th)^a^	No. (%)	BPb GM (5th–95th)^a^	No. (%)	BPb GM (5th–95th)^a^	*p*-Value^b^	*p*-Value^c^
Sex								
Male	83 (56.8)		12 (48.0)		79 (52.7)		0.49^d^	0.67^d^
Female	63 (43.2)		13 (52.0)		71 (47.3)			
SES								
Lowest	60 (42.9)		3 (12.0)		20 (13.3)		< 0.001^d^	0.82^d^
Medium	77 (55.0)		16 (64.0)		93 (62.0)			
Highest	3 (2.1)		6 (24.0)		37 (24.7)			
Apgar 5 min								
6	1 (0.7)		0 (0.0)		1 (0.7)		0.98^d^	1.00^d^
7	1 (0.7)		0 (0.0)		0 (0.0)			
8	8 (5.6)		1 (4.0)		9 (6.0)			
9	134 (93.1)		24 (96.0)		139 (92.6)			
10	0 (0.0)		0 (0.0)		1 (0.7)			
Birth order								
1	62 (42.8)		11 (44.0)		73 (48.7)		0.42^d^	0.87^d^
2	49 (33.8)		9 (36.0)		48 (32.0)			
3	28 (19.3)		3 (12.0)		22 (14.7)			
4	4 (2.8)		2 (8.0)		7 (4.7)			
≥ 5	2 (1.3)		0 (0.0)		0 (0.0)			
Birth weight (g)	144	3,194 (2,503–4,000)	25	3,148 (2,569–3,818)	150	3,218 (2,450–3,911)	0.64	0.46
Maternal IQ	127	91 (68–113)	25	96 (65–115)	150	93 (71–112)	0.31	0.26
Prenatal lead								
12–20 weeks	126	8.4 (7.6–9.1)	10	8.2 (3.0–13.7)	150	8.2 (3.0–20.7)	0.20	0.98
28–36 weeks	129	7.3 (1.5–17.4)	11	13.0 (5.3–27.0)	150	7.8 (2.5–24.6)	0.49	0.02
Bayley Scales of Infant Development								
MDI at 6 months	122	115.0 (91–140)	20	117.4 (98–137)	135	115.3 (89–144)	0.87	0.60
MDI at 12 months	103	114.1 (94–134)	25	115.2 (86–131)	137	115.7 (93–134)	0.33	0.85
MDI at 18 months	87	104.3 (78–128)	23	112.3 (102–128)	139	107.9 (88–128)	0.05	0.11
MDI at 24 months	69	103.5 (79–132)	21	119.8 (94–150)	135	109.5 (87–132)	0.009	0.003
McCarthy Scale								
GCI at 36 months	37	97.6 (64–117)	22	102.0 (89–118)	133	100.8 (85–116)	0.11	0.61
GCI at 42 months	25	98.7 (82–118)	20	110.1 (101–122)	133	105.1 (86–121)	0.01	0.06
GCI at 48 months	30	93.8 (60–130)	24	105.5 (85–119)	137	102.3 (81–122)	0.003	0.26
GCI at 54 months	18	95.8 (57–129)	22	106.8 (88–120)	124	104.1 (89–119)	0.008	0.29
GCI at 60 months	15	98.9 (62–127)	18	108.8 (82–121)	126	104.8 (88–119)	0.048	0.12
WISC FSIQ (years of age)								
6			23	109 (91–126)	140	105 (87–123)		0.17
7			20	109 (88–127)	140	109 (91–127)		0.93
8			21	109 (90–130)	127	108 (91–126)		0.72
9			16	114 (98–141)	120	109 (91–128)		0.09
10			15	112 (94–140)	115	109 (87–130)		0.45
Postnatal lead (years of age)								
1	131	10.0 (3.2–18.8)	23	11.6 (5.5–19.8)	142	10.8 (4.0–22.0)	0.40	0.54
2	93	12.0 (4.2–25.2)	25	13.1 (5.8–23.0)	142	12.8 (5.0–25.8)	0.42	0.82
3	52	11.6 (5.0–23.5)	25	12.2 (5.2–19.8)	140	11.3 (4.7–22.9)	0.74	0.52
4	38	8.9 (3.2–18.5)	25	11.3 (4.8–19.0)	142	10.3 (4.2–20.5)	0.13	0.46
5	22	9.0 (3.5–16.5)	22	10.6 (5.0–19.2)	136	9.3 (3.8–18.0)	0.78	0.26
6			21	9.3 (4.5–20.8)	135	7.9 (3.2–16.0)		0.14
7			21	8.9 (4.2–17.0)	142	7.5 (3.0–13.8)		0.13
8			20	7.5 (2.5–14.6)	132	6.4 (2.8–12.8)		0.17
9			21	7.7 (3.5–12.5)	123	6.0 (2.8–11.8)		0.025
10			15	7.8 (3.0–19.2)	118	5.6 (2.5–11.2)		0.008

Abreviations: GCI, General Cognitive Index; GM, geometric mean; MDI, Mental Development Index.

a Percentiles.

b Subjects included in the model versus subjects not assessed beyond 5 years of age.

c Subjects included in the model versus subjects with incomplete data.

d Fisher’s exact test of Pearson chi-square exact probability. Unmarked probabilities by *t*-test for independent samples.

**Table 2:** Nonadjusted and adjusted models of FSIQ (panel regressions).

	Unadjusted				Adjusted^a^		
Lead variable (μg/dL)	No.	β	95% CI	*p*-Value (two-tailed)	β_adj_	95% CI	*p*-Value (two-tailed)
Ln(lead) 12–20 weeks	150	−1.90	−4.79 to 0.98	0.20	−1.45	−4.75 to 2.00	0.42
Ln(lead) 28–36 weeks	150	−3.84	−6.24 to −1.44	0.002	−4.00	−6.37 to −1.65	0.001
Maternal ln(lead) delivery	112	−1.77	−5.12 to 1.57	0.29	−1.29	−4.41 to 1.83	0.41
Umbilical cord ln(lead)	109	−0.69	−3.50 to 2.11	0.63	−0.95	−3.65 to 1.75	0.49
Mean ln(lead) (1–5 years)	150	−2.41	−6.38 to 1.57	0.23	0.49	−3.81 to 4.81	0.82
Ln(lead) at 1 year of age	142	−1.51	−4.96 to 1.94	0.39	0.51	−3.19 to 4.21	0.79
Ln(lead) at 2 years of age	143	−1.10	−4.49 to 2.29	0.39	0.91	−2.67 to 4.49	0.62
Ln(lead) at 3 years of age	140	−2.53	−6.22 to 1.15	0.18	−0.58	−4.53 to 3.37	0.78
Ln(lead) at 4 years of age	142	−0.61	−4.34 to 3.12	0.75	1.17	−2.67 to 5.02	0.55
Ln(lead) at 5 years of age	136	−2.96	−6.67 to 0.75	0.12	−0.32	−4.26 to 3.36	0.87
Mean ln(lead) (1–2 years)	147	−1.78	−5.46 to 1.90	0.34	0.60	−3.36 to 4.57	0.76
Mean ln(lead) (3–5 years)	150	−2.63	−6.47 to 1.22	0.18	−0.08	−4.15 to 3.98	0.96
Mean ln(lead) (6–10 years)	150	−2.70	−4.23 to −1.16	0.001	−2.45	−4.09 to −0.81	0.003

CI, confidence interval. Each lead variable was tested alone.

a Adjusted by maternal IQ, SES, sex, birth weight, an indicator variable of first FSIQ application at 6, 7, or 8 years.

**Table 3:** Linear mixed models of FSIQ with random intercept and random slope for 6–10 year BPb (μg/dL): fixed-effects estimations.

	Model A (full model)			Model B (without nonsignificant control variables)			Model C (model B without nonsignificant lead before 6–10 years)			Model D (without any nonsignificant variables)		
Variable	β	95% CI	*p*-Value^a^	β	95% CI	*p*-Value	β	95% CI	*p*-Value	β	95% CI	*p*-Value
Intercept	73.6	52.4 to 94.6	< 0.0001	73.6	56.9 to 90.4	< 0.0001	75.8	62.6 to 88.0	< 0.0001	76.3	63.7 to 88.9	< 0.0001
Mean ln(lead) 12–20 weeks pregnancy (μg/dL)	1.02	−1.98 to 4.03	0.50	0.89	−2.09 to 3.88	0.56						
Mean ln(lead) 28–36 weeks pregnancy (μg/dL)	−3.90	−6.45 to −1.36	0.0029	−3.85	−6.36 to −1.33	0.0029	−3.46	−5.64 to −1.29	0.0020	−3.44	−5.61 to −1.28	0.0020
Mean ln(lead) 1–5 years (μg/dL)	0.10	−3.88 to 4.06	0.96	0.35	−3.48 to 4.18	0.86						
Ln(lead) 6–10 years (μg/dL)	0.17	−1.41 to 1.76	0.83	0.15	−1.44 to 1.72	0.86	0.21	−1.30 to 1.72	0.79			
Child sex (female)	−1.51	−4.75 to 1.73	0.36									
Birth weight (g)	0.001	−0.003 to 0.004	0.61									
SES (tertiles)	−0.38	−1.86 to 1.10	0.61									
Maternal IQ	0.40	0.26 to 0.55	< 0.0001	0.39	0.26 to 0.52	< 0.0001	0.39	0.26 to 0.51	< 0.0001	0.38	0.26 to 0.51	< 0.0001
First FSIQ measurement	−4.00	−4.84 to −3.16	< 0.0001	−4.00	−4.82 to −3.15	< 0.0001	−4.00	−4.83 to −3.16	< 0.0001	−4.00	−4.78 to −3.16	< 0.0001

CI, confidence interval.

a Two-tailed.

**Table 4:** Linear mixed model of FSIQ with random intercept and random slope for concurrent lead (*n* = 122) Test of prenatal lead concentration at week 28 of pregnancy: fixed-effects estimations.

Variable	β	95% CI	*p*-Value (two-tailed)
Intercept	79.5	56.5 to 102.5	< 0.0001
Ln(lead) 28 weeks pregnancy (μg/dL)	−4.13	−6.45 to −1.81	0.0006
Mean ln(lead) 1–5 years (μg/dL)	−1.01	−5.54 to 3.52	0.66
Ln(lead) 6–10 years (μg/dL)	0.21	−1.46 to 1.88	0.81
Child sex (female)	−1.21	−4.87 to 2.45	0.52
Birth weight (g)	0.001	−0.003 to 0.005	0.61
SES (tertiles)	−0.40	−1.27 to 2.07	0.64
Maternal IQ	0.38	0.22 to 0.54	< 0.0001
First FSIQ application	−3.52	−4.43 to −2.61	< 0.0001

CI, confidence interval.

## Appendix

**Appendix:** Linear mixed models with random intercept and random slope for concurrent lead for a cohort of 128 children:^a^fixed-effects estimations.

	Model A HOME and SES		Model B HOME without SES	
Variable	β ± SE (95% CI)	*p*-Value	β ± SE (95% CI)	*p*-Value
Intercept	75.09 ± 12.84 (49.80 to 100.38)	< 0.0001	75.18 ± 12.76 (50.04 to 100.32)	< 0.0001
Ln(lead) 12–20 weeks	0.90 ± 1.640 (−2.34 to 4.12)	0.5864	0.88 ± 1.63 (−2.33 to 4.09)	0.5892
Ln(lead) 28–38 weeks	−4.15 ± 1.34 (−6.79 to −1.51)	0.0024	−4.14 ± 1.33 (−6.76 to −1.52)	0.0023
Mean ln(lead) 1–5 years	0.56 ± 2.22 (−3.81 to 4.93)	0.8013	0.61 ± 2.14 (−3.61 to 4.83)	0.7746
Ln(lead) 6–10 years	0.46 ± 0.85 (−1.21 to 2.13)	0.5907	0.46 ± 0.85 (−1.21 to 2.13)	0.5911
Child sex	−0.64 ± 1.83 (−4.25 to 2.97)	0.7270	−0.67 ± 1.80 (−4.22 to 2.88)	0.7109
Birth weight	0.00 ± 0.02 (−0.004 to 0.004)	0.9070	0.00023 ± 0.002 (−0.004, to 0.004)	0.9076
SES	−0.10 ± 0.86 (−2.59 to 0.79)	0.9170		
HOME scale	−0.16 ± 0.15 (−0.46 to 0.14)	0.3103	−0.16 ± 0.15 (−0.46 to 0.14)	0.2686
Maternal IQ	0.42 ± 0.08 (0.26 to 0.58)	< 0.0001	0.42 ± 0.07 (0.28 to 0.56)	< 0.0001
First FSIQ application indicator	−4.11 ± 0.45 (3.20 to 5.02)	< 0.0001	−4.11 ± 0.46 (−5.02 to −3.20)	< 0.0001

CI, confidence interval.

a Dependent variable is FSIQ of children from 6 to 10 years of age.
